# Mechanical Properties and Ion Release from Fibre-Reinforced Glass Ionomer Cement

**DOI:** 10.3390/polym16050607

**Published:** 2024-02-23

**Authors:** Anja Ivica, Ivan Šalinović, Silvana Jukić Krmek, Sufyan Garoushi, Lippo Lassila, Eija Säilynoja, Ivana Miletić

**Affiliations:** 1 Department of Endodontics and Restorative Dentistry, School of Dental Medicine, University of Zagreb, 10000 Zagreb, Croatia; aivica@sfzg.hr (A.I.); jukic@sfzg.hr (S.J.K.); miletic@sfzg.hr (I.M.); 2 Department of Biomaterials Science and Turku Clinical Biomaterial Center—TCBC Institute of Dentistry, University of Turku, 20520 Turku, Finland; liplas@utu.fi (L.L.); eija.sailynoja@gc.dental (E.S.); 3 Research Development and Production Department, Stick Tech Ltd., 20520 Turku, Finland

**Keywords:** glass fibres, glass ionomer cements, fluoride release, reinforcement

## Abstract

The aim of this study was to compare the mechanical properties and ion release from a commercially available resin-modified glass ionomer cement to a formulation reinforced by the addition of short glass fibres at various percentages. **Methods:** Three experimental groups were prepared by adding a mass ratio of 10%, 15% and 20% of short glass fibres to the powder portion of the cement from a capsule (GC Fuji II LC), while the control group contained no fibres. Microhardness (n = 12), fracture toughness, and flexural, compressive and diametral tensile strength (n = 8) were evaluated. To study ion release, readings were obtained utilising fluoro-selective and calcium-selective electrodes after 24 h, 7 days and 30 days (n = 12). The spatial distribution of fibres within the material was evaluated through scanning electron microscopy. The data were analysed using one-way ANOVA with a Bonferroni adjustment. **Results:** The findings suggest that elevating fibre weight ratios to 20 wt% results in improved mechanical properties (*p* < 0.05) in microhardness, flexural strength, diametral tensile strength and fracture toughness. In terms of ion release, a statistically significant difference (*p* < 0.001) was observed between the groups at the conclusion of 24 h and 7 days, when the fluoride release was much higher in the control group. However, after 30 days, no significant distinction among the groups was identified (*p* > 0.05). Regarding calcium release, no statistically significant differences were observed among the groups at any of the evaluated time points (*p* > 0.05). SEM showed the fibres were homogeneously incorporated into the cement in all experimental groups. **Conclusions:** Resin-modified glass ionomer enhanced with short glass fibres at a weight loading of 20% showcased the most favourable mechanical properties while concurrently maintaining the ability to release fluoride and calcium after a 30-day period.

## 1. Introduction

Glass ionomer cements (GICs) are one of the most commonly used dental restorative materials [1]. However, GIC restorations have significantly shorter longevity compared to resin composites, which is why they are mainly used as temporary restorations [2]. The main reasons for failure seem to be the loss of anatomical contour and the loss of proximal contacts, which can be explained by the fact that their mechanical strength is relatively low compared to resin composites [3]. There are numerous advantages of GIC materials over other restorative materials [4]. Nevertheless, GICs were the first dental restorative materials to exhibit bioactive properties [1]. They release fluoride into the oral environment, and the technique for applying GIC is usually faster compared to resin composites [5]. The restorative technique for resin composite placement is very sensitive and requires a greater number of operative steps. Therefore, in cases in which the use of a rubber dam is problematic or patient compliance is limited, GIC is the gold standard [6]. For the same reasons, conventional GIC and resin-modified GIC are the preferred materials for cervical lesions [7]. Although resin-modified GIC represents significant progress in mechanical properties compared to conventional GICs, the desired mechanical properties have not been reached [8].

A restorative material must have sufficient strength and stability against occlusal loading, thermal changes and other influences in the oral environment to be clinically applicable in posterior dentition [9]. The poor mechanical properties of GIC materials, such as low fracture toughness, flexural strength and microhardness, limit their extensive use in dentistry as a filling material in stress-bearing areas [4,9]. In the posterior area, GICs are mostly used as a temporary filling material. The requirement to strengthen GICs has led to an ever-increasing research effort into reinforcement techniques. Despite the various improvements in both powder and liquid formulations, further enhancement of the mechanical properties of GIC materials is needed [10,11]. The fibre reinforcement approach has been successfully adopted and used to reinforce resin composites [12]. The concept of fibre reinforcement in dentistry aims to enhance the internal strength of a structurally compromised tooth, minimising the risk of fractures [13].

Two distinct factors seem to be critical for the clinical efficacy of GIC materials: the release of ions [14] and the mechanical properties [15]. Although the minimum local concentration of fluoride release required to inhibit the progression of caries has not been determined, reinforcement with various particles alters the composition of GIC and may affect the potential for ion release [16]. Hence, the goals of the present study were to identify the material with improved mechanical properties without compromising the release potential of fluoride and calcium ions.

The null-hypotheses were: (I) there is no difference between tested materials in any of the examined parameters; and (II) for any given parameter, there is no difference at different time points.

## 2. Materials and Methods

### 2.1. Examined Materials and Preparation of Samples

In the present investigation, a radiopaque light-cured encapsulated GIC, namely Fuji II LC (GC Corporation Tokyo, Japan), with each capsule containing 0.33 g of powder and 0.1 g (0.085 mL) of liquid, was used. The samples were prepared according to the ISO 4049:2010 standard [17]. Three experimental groups were prepared by carefully opening the capsule and 10%, 15% or 20% weight of powder was exchanged with short glass fibres (Central Glass Fiber Co., Ltd., Tokyo, Japan), with a 6 µm diameter and an average length of 140 µm. Prior to amalgamation with the liquid component, the glass fibres were thoroughly mixed with the GIC powder. The control group was devoid of any fibre inclusion. Following this preparation, the capsule underwent activation and mixing in 3M™ ESPE™ CapMixTM (3M ESPE, Seefeld, Germany) for 10 s, according to the manufacturer’s specified instructions.

### 2.2. Microhardness

For the microhardness assessment, the specimens (n = 24) were meticulously prepared using circular Teflon moulds with dimensions of 2 mm in height and 5 mm in width according to ISO 4049 [17]. These moulds were positioned on a glass tile covered with acetate foil and subsequently filled with the GIC under investigation and covered with acetate foil on the opposing side. After the curing process of 20 s using Bluephase G2 (Ivoclar Vivadent, Schaan, Liechtenstein), with 950 mW/cm^2^, the acetate foil and excess material were carefully removed. Following the hardening process, half of the samples were promptly evaluated, while the remaining specimens (n = 12 per group) underwent a 30-day immersion in vials (Laboroprema, Zagreb, Croatia) with 5 mL of deionised water within an ES 120 incubator (Nüve, Ankara, Turkey) set at a constant temperature of 37 °C. Microhardness measurements were conducted utilising the Qness—Q10 M—Microhardness Tester (ATM Qness GmbH, Golling an der Salzach, Austria). The Vickers method, specifically corresponding to the HV0.1 technique, was employed with a 10-s application of a 100 g load. Three readings were taken for each sample, with the arithmetic mean recorded as the accepted value. Notably, the distance between indents was maintained at least three times their diameter.

### 2.3. Flexural Strength

The flexural strength (FS) was assessed by subjecting bar-shaped specimens (2 × 2 × 25 mm^3^ as proposed in ISO 4049 [17]) made from each tested material (n = 8 per group). The specimens were fabricated within a semi-split Teflon mould positioned between transparent Mylar sheets and a glass slide. Resin-modified GIC specimens underwent polymerisation using a hand light-curing unit for 20 s in five distinct overlapping sections from both sides of the Teflon mould. Subsequently, specimens from each group were stored in a wet environment at 37 °C for 24 h before testing. The three-point bending test, in accordance with ISO 4049, was executed on all specimens using a material testing machine (model LRX, Lloyd Instruments Ltd., Fareham, England). The flexural strength (ơ_f_) was computed using the following equation based on ISO 1992 standards [18]:

ơ_f_ = 3F_m_I/2bh^2^ (1)

Here, F_m_ represents the applied load (N) at the peak of a load-deflection curve, I is the span length (20 mm), b denotes the width of the test specimens and h is the thickness of the test specimens.

### 2.4. Compressive and Diametral Tensile Strengths

Compressive strength (CS) and diametral tensile strength (DTS) measurements were conducted on cylindrical specimens with a diameter of 4 mm and a height of 6 mm, which were prepared following the procedures outlined in ISO 9917 [19]. For the CS evaluation, identical testing procedures were employed, involving the placement of specimens (n = 8 per group) with their flat ends on the supporting plate. The flat ends were securely positioned between the platens of the testing machine (Lloyd). In this scenario, a compressive load was applied axially until failure occurred at a crosshead speed of 1 mm/min. The length and diameter of each specimen were measured before testing with a digital calliper. The CS was determined by assessing the peak load at fracture and the diameter of the specimen. The CS was calculated in megapascals (MPa) using the equation:

P = F/D(2)

where P is the compressive strength, F is the maximum applied load in newtons (N) and D is the cross-sectional area of the specimen.

For the DTS evaluation, each specimen was positioned with its longitudinal side situated between the platens of the testing machine. The specimens (n = 8 per group) underwent compressive loading until failure occurred. The DTS was calculated based on the specimen length, diameter and peak load. The DTS in megapascals (MPa) was calculated using the equation:

T = 2F/π lD(3)

where T is the strength, F is the maximum applied load in newtons (N), D is the diameter of the specimens in mm and l is the length of the specimen in mm.

### 2.5. Fracture Toughness

Specimens in the form of single-edge notched beams (2.5 × 5 × 25 mm³) were prepared following the previously used method [18,20,21] to determine fracture toughness (FT). A custom-made Teflon split mould, facilitating easy specimen removal without force, was utilised. The mould featured a centrally located, accurately designed slot extending to its mid-height, optimising the crack length to be 0.5. The GIC material was placed into the mould, positioned over a Mylar-strip-covered glass slide, in a single increment. Before setting and polymerisation, a sharp, centrally located crack was created by inserting a straight-edged steel blade into the prefabricated slot. Polymerisation of the GIC occurred for 20 s in five separate overlapping portions. The upper side of the mould was covered with a Mylar strip and a glass slide on both sides of the blade before exposure to the polymerisation light. After removal from the mould, each specimen was polymerised on the opposite side as well. Specimens from each group (n = 8) were stored wet at 37 °C for 24 h before testing. The testing involved three-point bending mode, conducted in a universal material testing machine with a crosshead speed of 1.0 mm/min.

### 2.6. Fluoride and Calcium Release

For the analysis of ion release, specimens (n = 12) were prepared using circular Teflon moulds with dimensions of 2 mm in height and 8 mm in width as described in ISO specification 9917 [19].

Subsequent to the curing process, the samples were stored in vials (Laboroprema) with 5 mL of deionised water at 37 °C in an incubator ES 120 (Nüve, Ankara, Turkey) for 24 h, 7 days and 30 days, respectively.

The analysis of the fluoride and calcium concentrations in aqueous solutions was performed according to the ISO 19448:2018 standard [22]. 

The fluoride concentrations in the water samples were quantified utilising an ion-selective electrode (F800 DIN, Xylem Analytics Germany, Weilheim, Germany) coupled with an ion analyser (inoLab Multi 9630 DS; Xylem Analytics Germany, Weilheim, Germany). The calibration of the electrode was performed daily using a series of standard solutions (20 µM/L NaF, 50 µM/L NaF, and 100 µM/L NaF). Subsequent to each measurement, the electrode was rinsed with fresh deionised water and dried with absorbent paper. A 5 mL aliquot of immersion media was mixed with an additional 5 mL of a total ionic strength adjustment buffer (Merck KGaA, Darmstadt, Germany), and the fluoride concentrations were determined after a 5-min incubation period. During the readings, the specimens were extracted from the vials, dried with absorbent paper and transferred to a new vial containing fresh distilled water. Each measurement was conducted in triplicate, and the results were expressed in mg/L (ppm F−).

The calcium release was determined using a calcium-selective electrode Ca 800 DIN (Xylem Analytics Germany GmbH, Weilheim, Germany). Before measuring, the samples were removed from deionised water and 2% of an ISA/Ca solution (WTW, Weilheim, Germany) was added. For each sample, three reading were taken and the mean value was calculated and taken as a result. The results were expressed in milligrams per litre (mg/L).

### 2.7. Scanning Electron Microscopy

To visualise the microstructure of the test and the control GIC, triplicates of the specimens were inspected using scanning electron microscopy (SEM). To this end, the samples were attached to sticky carbon pads (Plano, Wetzlar, Germany) on SEM pin stubs. The specimens were sputtered with an 8 nm gold layer (Safematic, Bad Ragaz, Switzerland). Images were obtained at a 5 kV acceleration voltage with the vacuum set to 200 Pa, a 1000 times magnification, and a 10 mm working distance using a secondary electron detector (Zeiss Supra 50VP, Oberkochen, Germany).

### 2.8. Statistical Analysis

All experiments were repeated at least three times independently to verify reproducibility. GraphPad Prism version 9.20 was used for the statistical evaluation (GraphPad, La Jolla, CA, USA). The data were expressed as mean ± standard deviation. A one-way analysis of variance (ANOVA) was used to determine whether there were any statistically significant differences among the groups. A Bonferroni adjustment was applied for multiple comparisons. The level of statistical significance was set at *p* < 0.05.

## 3. Results

### 3.1. Microhardness

**Figure 1:**
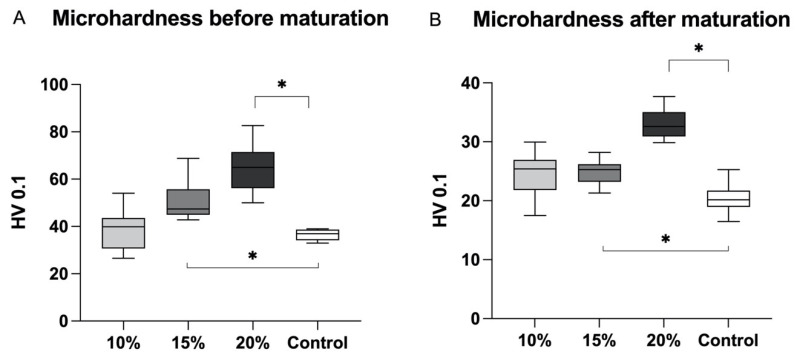
Microhardness quantification was performed promptly after sample preparation (**A**) and following a 30-day maturation period (**B**). The GIC fortified with 20% of glass fibres demonstrated the highest hardness among the tested groups, exhibiting a statistical significance (*p* < 0.001). Mean values were expressed together with maximal and minimal obtained values. Asterisk (∗) indicates level of statistical significance *p* ≤ 0.05.

Figure 1 illustrates the microhardness results pre- and post-maturation. At both time points, it is evident that the GIC with 20% of glass fibres displayed a significantly harder surface (*p* < 0.001) compared to the control and test groups reinforced with 15% and 10% of glass fibres.

The results indicate that, across all tested groups, the microhardness values were lower after a 30-day maturation period compared to the initial measurements.

### 3.2. Flexural Strength, Compressive Strength, Diametral Tensile Strength and Fracture Toughness

The average values of flexural strength (FS), compressive strength (CS), diametral tensile strength (DTS) and fracture toughness (FT) for the tested materials, along with their standard deviations (SD), are presented in Table 1. Generally, the incorporation of short fibres resulted in a significant improvement in most of the mechanical properties of resin-modified GICs (*p* < 0.05). The data indicate that an increase in fibre weight ratios to 20 wt% led to an enhancement in the mechanical properties (*p* < 0.05). However, compressive strength was the exception, showing no improvement; the unmodified commercial GIC exhibited a higher CS (191 MPa) compared to all other experimental GIC materials. Tukey HSD post hoc analysis revealed that the GIC with 20 wt% of fibres had statistically significantly higher FT values (1.7 MPam^1/2^) compared to all other tested GIC materials.

### 3.3. Fluoride Release

**Figure 2:**
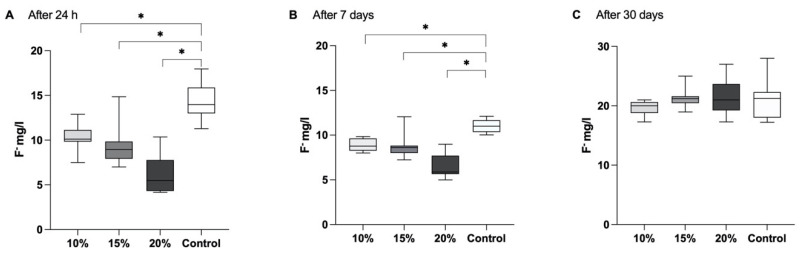
Mean values with minimum and maximum obtained values were expressed for different time points. After 24 h (**A**) the highest fluoride release was exhibited by the control material, followed by the material reinforced with 10% of glass fibres, then 15%, and, finally, 20% (*p* < 0.001). The control group exhibited a statistically significant increase in fluoride release when compared to the tested materials. Similar results were obtained after 7 days (**B**). The findings concerning fluoride release after a 30-day period (**C**) indicated detectable levels in all groups, and no statistically significant differences were observed among the groups (*p* > 0.05). Asterisk (∗) indicates level of statistical significance *p* ≤ 0.05.

A substantial release of fluoride ions was observed and quantifiable across all groups after 24 h (Figure 2). The control material manifested the highest fluoride release, succeeded by the material reinforced with 10% of glass fibres, then 15% and, finally, 20% (*p* < 0.001). The values obtained after 24 h are as follows: 10.38 ± 1.39 for the 10% group; 9.36 ± 2.05 for the 15% group; 6.13 ± 2.04 for the 15% group and 14.36 ± 1.96 for the control.

A similar trend in fluoride release was evident after 7 days, with a noteworthy distinction observed between the control group and the experimental groups at this temporal point (*p* < 0.01). At this time point, the following values were obtained: 8.85 ± 0.69 for the 10% group; 8.77 ± 1.43 for the 15% group; 6.49 ± 1.36 for the 20% group and 11.03 ± 0.72 for the control.

The results pertaining to fluoride release after 30 days revealed detectable levels across all groups, with no significant differences identified between the groups (*p* > 0.05).

The values acquired after a 30-days period are as follows: 16.69 ± 1.24 for the 10% group; 21.29 ± 1.64 for the 15% group; 21.41 ± 2.96 for the 20% group and 21.15 ± 3.26 for the control.

### 3.4. Calcium Release

**Figure 3:**
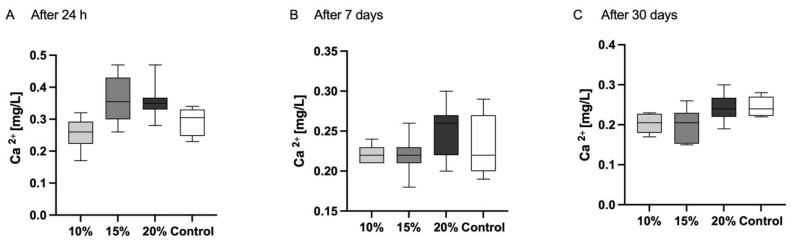
For various time points, the mean values along with corresponding minimum and maximum values are presented. The highest values of calcium release occurred in the first 24 h for all tested GIC materials (**A**). There was no statistical difference in cumulative calcium release over the period of 24 h (**A**), 7 days (**B**) or 30 days (**C**) among different groups.

A quantifiable release of calcium ions was observed consistently across all groups at 24 h, 7 days and 30 days (Figure 3). However, there were no statistical differences observed among groups (*p* > 0.05).

### 3.5. SEM

**Figure 4:**
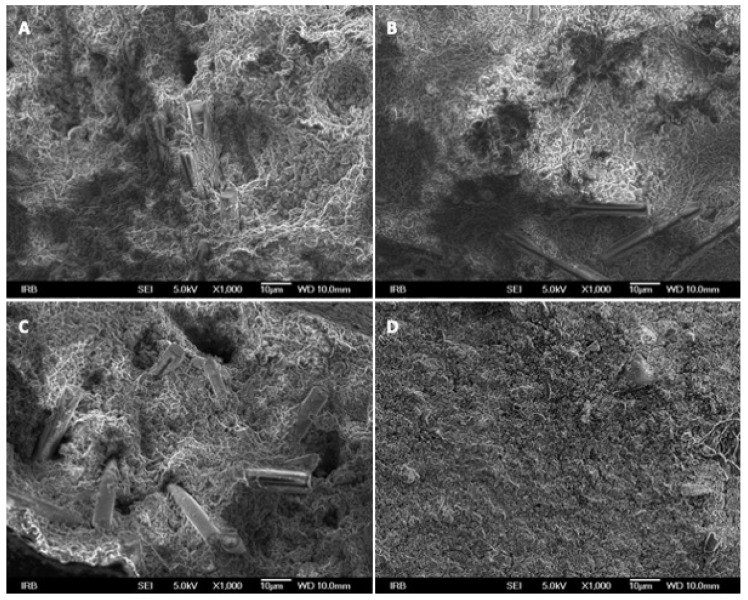
SEM was used to study the structure of resin-modified GIC reinforced with different proportions of glass fibres: 10% of glass fibres (**A**), 15% (**B**), 20% (**C**) and without glass fibres (**D**).

Figure 4 presents details on the microstructure of the different test materials with different reinforcements (A–C) and the control material without glass fibres (D) investigated in this study. Glass fibres are seen in panels A–C. In reinforced materials, glass fibres are randomly distributed among the base material.

## 4. Discussion

Microhardness, flexural strength, compressive strength, diametral tensile strength, fracture toughness and fluoride release are crucial parameters for evaluating a restorative material according to the consensus on GIC thresholds for restorative indications [23]. In this study, different proportions of glass fibres were tested to determine their influence on these properties when incorporated into resin-modified GIC.

The results showed that the use of short glass fibre fillers within a resin-modified GIC matrix yielded a superior mechanical performance compared to the control group. Therefore, the first null hypothesis was fully rejected. Nevertheless, the tested materials were able to release fluoride and calcium in a similar manner as the control, so the second null hypothesis was partially rejected.

Samples with more than 20% of fibres were not included because it was previously found that it was not possible to mix the material properly with a higher percentage of fibre mass [24]. According to Dowling et al., the optimum fibre length was calculated to be 50 times the fibre diameter [25]. However, since the fibres are damaged during mixing, the desired fibre length is usually not achieved in the tested materials [24] and in the present study the diameter was 6 μm, while the fibre length was 140 μm, which did not alter the mixing procedure. Scanning electron microscopy (SEM) analysis of all tested combinations involving glass fibres revealed a homogeneous structure.

Fuji II was selected as the GIC base material because it contains the monomer 2-hydroxyethyl methacrylate (HEMA), which promotes both hydrogen and covalent bonding, crucial for establishing strong and durable adhesion between the fibres and the matrix of the cement [26]. Effective adhesion between the fibre and the matrix facilitates an efficient load transfer, ensuring that the load is effectively transmitted to the stronger fibre, enhancing its role as reinforcement. Insufficient adhesion and the presence of voids between the fibre and the RMGIC matrix can serve as potential fracture sites in the matrix, leading to material breakdown [27]. The experimental resin-modified GIC demonstrated effective wetting of the microfibers by the matrix, which can be attributed to the favourable reinforcing effect observed. The fibres in this investigation were obtained from the manufacturer and already treated with a silane coating. The specific silane employed is 3-(Trimethoxysilyl) propyl methacrylate (MPS), which is compatible with acrylate resins. When polyacrylic acid esterifies the surface of fibre with silane, it forms a polymer that contains functional double bonds [21]. The gradual release of ions through an acid attack on the glass surface is followed by temporal alterations in structure [28]. Prior research has indicated that the existence of aluminium and calcium ions on the surface of glass fibres enables the formation of a reactive layer at the junction between the glass fibre surface and polyacrylic acid [21]. This bond is a critical factor in stress transfer [29]. It has been reported that the use of a resin-modified GIC gives better results compared to conventional GIC [8,26] because the matrix of conventional GIC cannot provide sufficient mechanical strength and compatibility for effective fibre reinforcement. However, the results of Hammouda et al. demonstrated that the reinforcement of conventional GIC with 3 wt% and 5 wt% of short glass fibres showed better mechanical properties compared with the control group. In this study, the results for microhardness in the tested groups are much higher compared to those results [30]. This could be explained by the present study using larger amounts of glass fibres. It is also possible, as stated before, that the resin matrix in RMGIC can form stronger bonds with both the glass particles and the fibres.

The microhardness was assessed at two distinct time intervals: immediately following sample fabrication and after a 30-day period of maturation in distilled water. The findings consistently exhibited a recurring pattern, with the highest microhardness values being observed in the group containing 20 wt% of glass fibres. However, the samples subjected to a 30-day immersion in water displayed slightly reduced microhardness values compared to their pre-maturation measurements. This phenomenon can be attributed to their prolonged exposure to water, resulting in the release of ions, which may lead to alterations in the material’s mechanical properties [31]. Prior research had previously noted that the microhardness in GIC samples aged for six months was marginally lower than that in samples aged for three months, though this discrepancy did not achieve statistical significance [32]. When conventional GIC is exposed to water before the formation of calcium and aluminium polyacrylate salts, there is a risk of losing calcium and aluminium ions, significantly impacting the material’s mechanical characteristics. However, resin-modified GIC incorporates HEMA, which initiates the setting reaction of GIC, while the acid-base glass-ionomer reactions continue beneath, preventing the loss of ions [33].

In the realm of mechanical properties, compressive strength was the only parameter that did not exhibit any notable improvement between the fibre-reinforced and unmodified commercial GIC, aligning with findings from similar studies [18]. This can be partially attributed to the specimen’s geometry and test setup, as the literature highlights the importance of fibre orientation relative to the load direction in influencing the mechanical properties of fibre-reinforced composites [21]. Dowling et al. recommend against advocating for compressive fracture strength in predicting the performance of GIC materials [34]. This is in line with the a recent systematic review [35] showing that fracture toughness of dental composites had a direct correlation with clinical success. The fracture toughness of fibre-reinforced GIC groups (1.7 MPam^1/2^) is at the same level as that of commercial particulate-filled flowable composite materials [36]. As a consequence, improvements in the material’s ability to resist fatigue crack propagation, as well as increased fracture energy and toughness of restoration, can be expected [37].

In a previous study it was observed that the control GIC exhibited the highest fluoride release among the materials tested [38], which was consistent with these results. This may be attributed to the increased presence of fibres within the material, resulting in a reduced matrix material proportion and, consequently, a diminished fluoride release. SEM analysis revealed a homogeneous distribution of fibres on the surface, resulting in a reduction of the surface area of the base GIC material capable of ion release. Moreover, in the groups where modified GIC was used, a notable release of fluoride and calcium ions persisted after a 30-day duration, indicative of the persistent caries-protective attributes, even in the presence of a 20% fibre augmentation within the resin-modified GIC. This aligns with findings from other investigations on the inclusion of different particles in GIC [39,40]. This observation can be elucidated by considering the diffusion pathways inherent in the base material. Within all experimental cohorts in the present study, a peak in ion release was discerned during the initial hours subsequent to immersing the samples in deionised water, followed by a gradual diminishment of ion release. This pattern was similarly evident in various other media, including artificial saliva and lactic acid [41].

Apart from fluoride, the release of calcium and phosphates may induce the rebuilding of demineralised dentin by deposition of apatite-like crystals and, moreover, protect the collagen from enzymatic degradation [42]. In comparison to fluoride ions, the release of calcium ions occurs in substantially smaller quantities, as also observed by previous researchers [43]. It may be assumed that in an acidic environment, fluoride and calcium release would be greater, which is optimal for the protection of the tooth structure from demineralisation. However, a higher calcium release may correspond to alterations in the material’s mass and solubility [44,45]. Also, it was shown that the conventional GIC was more affected by an acidic environment when compared to resin-modified GIC [46].

This study has potential limitations. The present investigation focuses on the mechanical properties and ion release of a commercially available resin-modified GIC reinforced through the incorporation of short glass fibres in varying proportions, under in vitro conditions. Consequently, caution is needed when drawing clinical conclusions from the obtained data. Factors such as the oral environment, dietary influences, acidification during caries development, occlusal forces, moisture control during dental interventions and oral hygiene are acknowledged to potentially impact the mechanical characteristics of dental materials [6,47]. Given that water is essential for the adequate maturation of a GIC material, any contamination during the initial setting phases may undermine the physical characteristics of the restoration [48]. It is noteworthy to mention that enhancing the mechanical properties should not negatively impact the aesthetic properties of the material [49]. Additional clinical research is needed to examine the adhesion to dentin and the long-term prognosis of GIC reinforced with glass fibres. There are still unclear issues regarding the fibre’s interaction with the matrix and dispersion of the short glass fibres within the base material that need to be resolved. Therefore, to discern the optimal GIC suitable for stress-bearing areas as a long-term restorative material, further studies are needed.

## 5. Conclusions

Within the study’s limitations, it can be concluded that GIC reinforced with short glass fibres at a weight loading of 20% demonstrated the most favourable mechanical properties and, at the same time, preserved the capacity to release fluoride and calcium.

## Figures and Tables

**Table 1:** Mechanical properties mean values (±SD) of investigated materials.

Material	FS (MPa)	CS (MPa)	DTS (MPa)	FT (MPam^1/2^)
Control	52 (9) ^A^	191 (12) ^AB^	21 (3) ^A^	0.8 (0.1) ^A^
10%	60 (6) ^AB^	174 (8) ^B^	23 (2) ^AB^	1.4 (0.3) ^B^
15%	62 (10) ^AB^	173 (9) ^B^	25 (3) ^AB^	1.4 (0.2) ^B^
20%	78 (12) ^BC^	172 (18) ^B^	30 (4) ^BC^	1.7 (0.1) ^C^

FS: Flexural strength, CS: Compressive strength, DTS: diametral tensile strength FT: fracture toughness. Same superscript letter above the values indicates groups that were not statistically different (*p* > 0.05).

## Data Availability

Data are contained within the article.
